# Integrated Analysis of Single‐Cell and Bulk RNA‐Sequencing Defines N7‐Methylguanosine (m7G)‐Mediated Modifications' Role in Prognosis and the Tumor Immune Microenvironment in Hepatocellular Carcinoma

**DOI:** 10.1002/cam4.70992

**Published:** 2025-06-09

**Authors:** Jiahua Liang, Mingjian Ma, Borui Xu, Cheng Zhong, Guangyan Zeng, Huijiao Lu, Jiaming Lai, Jiancong Chen

**Affiliations:** ^1^ Department of Pancreato‐Biliary Surgery The First Affiliated Hospital, Sun Yat‐Sen University Guangzhou China; ^2^ Department of Orthopedics Jiangmen Hospital of Traditional Chinese Medicine Affiliated to Jinan University Jiangmen China; ^3^ Department of Gastrointestinal Surgery Eighth Affiliated Hospital, Sun Yat‐Sen University Shenzhen China; ^4^ School of Life Sciences, Sun Yat‐Sen University Guangzhou China

**Keywords:** hepatocellular carcinoma microenvironment, immune checkpoint inhibitors, m7G modification, single‐cell RNA sequencing, tumor immune microenvironment

## Abstract

**Background:**

RNA 7‐Methylguanosine (m7G) modification is biologically important to tumorigenesis and progression. Knowledge regarding the effect of m7G modification on the clinical features of hepatocellular carcinoma and its prognosis, tumor immune microenvironment, and sensitivity to immunotherapy remains limited.

**Method:**

Single‐cell RNA sequencing (scRNA‐seq) data was sourced from the GEO repository. Transcriptomic datasets were procured from TCGA, GEO, and ICGC databases. Following this, we scrutinized 17 m7G‐associated genes and executed unsupervised clustering to discern m7G modification profiles within HCC. Evaluation of immune cell infiltration levels and associated biological functions across varied m7G modification patterns was conducted using single‐sample gene set enrichment analysis (ssGSEA) complemented by gene set variation analysis (GSVA). In addition, we employed principal component analysis methodologies to elucidate m7G modification profiles in individual tumors and subsequently devised the m7Gscore. Data pertaining to m7G‐related protein and mRNA levels were sourced from the Human Protein Atlas (HPA) database. These findings were corroborated in both HCC and normal samples utilizing western blot analysis, immunohistochemical assays, and RT‐qPCR evaluations.

**Results:**

Our analysis unveiled diverse cellular subgroups within HCC, with a particular focus on T‐cell populations, providing insights into the intricate interplay of immune activation and suppression within the tumor microenvironment (TME). HCC patients were stratified into three distinct m7G modification patterns: immune‐desert, immune‐inflamed, and immune‐excluded. Subsequently, m7Gscores, derived from m7G‐associated signature genes, categorized HCC patients into high and low m7Gscore groups. Patients in the high‐score cohort exhibited significantly improved survival outcomes compared to those in the low‐score cohort. Elevated m7Gscores also correlated with enhanced therapeutic responsiveness, leading to improved outcomes in both chemotherapy and immunotherapy regimens. Our findings were further validated through RT‐qPCR, western blot analyses, and immunohistochemical assays, providing robust support for our bioinformatic observations.

**Conclusion:**

The m7G modification profiles play a pivotal role in prognosticating outcomes for HCC patients, intricately intertwined with microenvironmental heterogeneity and complexity. Furthermore, the analytical insights gained from the m7Gscore signature not only shed light on tumor microenvironment infiltration dynamics but also strengthen prognostic precision, paving the way for optimizing immunotherapeutic approaches in HCC.

**Abbreviations**
CN: copy number variants
DEG: differentially expressed gene
GSVA: Gene Set Variation Analysis
HCC: hepatocellular carcinoma
ICIA: immune checkpoint inhibitors
IPS: Immunophenoscore
m7G: 7‐Methylguanosine
ssGSEA: Single‐Sample Gene Set Enrichment Analysis
TCI: Cancer Immunome Atlas
TME: tumor microenvironment

## 1 Introduction

Hepatocellular carcinoma (HCC) represents the predominant form of liver malignancies, accounting for approximately 90% of cases, and stands as the fourth primary contributor to global cancer‐related mortality [1]. Although the mortality rate has stabilized due to treatment advances, the five‐year survival rate remains low, at approximately 20% [2]. Therefore, it is crucial to understand the pathogenesis of HCC and identify more effective treatment methods.

RNA modification regulates RNA metabolism, affects mRNA translation, and is closely associated with cancer development [3]. N7‐Methylguanosine (m7G) methylation, an emerging area of interest in the realm of RNA modifications, holds significant implications for various cellular activities, encompassing the dysregulation of mRNA, tRNA, and rRNA [4]. Human homologous METTL1 (methyltransferase‐like protein 1) and WDR4 (WD repeat domain 4) are crucial regulators in m7G tRNA modification [5]. Recent literature underscores a growing body of evidence suggesting that dysregulation of m7G‐associated genes could play a pivotal role in the oncogenesis of malignant neoplasms. As delineated by Peng Xia [6], the upregulation of WDR4 expression in HCC increases m7G methylation levels, and WDR4 is an important promoter of HCC development and progression. Additionally, Zhihang Chen [7] demonstrated that the m7G codon promotes the proliferation and metastasis of HCC in a frequency‐dependent manner. However, few studies have reported the regulatory mechanisms of m7G‐related genes and m7G RNA modification patterns in the development and progression of HCC [8].

The tumor microenvironment (TME), including endothelial cells, immune cells, mesenchymal cells, extracellular matrix, and cytokines secreted by these components, is the internal environment of the tumor that directly supports the survival and development of tumor cells [9]. An intricate comprehension of the dynamic interplay between the tumor microenvironment and immune response has substantially advanced the evolution of immunotherapeutic approaches [10]. Recent investigations have unveiled a notable association between immune cell infiltration within the tumor microenvironment (TME) and RNA modifications [11, 12, 13]. Nonetheless, insights into the interplay between m7G RNA modification and immune cell infiltration within the HCC tumor microenvironment (TME) are still in their infancy [14]. Elucidating the nuances of TME immune cell dynamics driven by m7G RNA modification could further our comprehension of TME immunoregulation.

In this investigation, harnessing transcriptomic and genomic data from 761 HCC specimens sourced from the Cancer Genome Atlas (TCGA) and Gene Expression Omnibus (GEO) repositories, we probed the nexus between m7G modification paradigms and characteristics of immune cells infiltrating the TME. Critical insights from single‐cell sequencing further illuminated the intricate cellular heterogeneity of HCC, often hidden in traditional bulk RNA sequencing approaches, emphasizing the nuanced roles that m7G‐related genes play within this cellular mosaic. Three distinct m7G modification patterns were discerned. We observed different immune statuses and prognoses among these modes, underlining the pivotal role m7G modification has in shaping TME characteristics in HCC. The scoring system we devised quantifies the m7G modification patterns of individual tumors and offers a predictive lens for patients' clinical responses to immune checkpoint inhibitor (ICI) therapy. Our findings highlight the significant influence m7G modification patterns have on crafting diverse tumor immune microenvironments, tumorigenesis, and progression in HCC, positioning these patterns as potent biomarker candidates for prognostic forecasting and therapeutic selection in HCC patients.

## 2 Materials and Methods

### 2.1 Acquisition of Hepatocellular Carcinoma Data Sources and Preprocessing

Data on gene expression levels and clinical characteristics (including patient survival status, overall survival time, age, sex, and tumor stage data) for hepatocellular carcinoma samples were retrieved retrospectively from the TCGA (https://cancergenome.nih.gov/) and NCBI GEO (https://www.ncbi.nlm.nih.gov/geo/) databases. Our subsequent analysis excluded patients without available information regarding their overall survival time. For further investigation, three relevant cohorts of hepatocellular carcinoma patients were obtained (GSE14520, GSE76427, and TCGA‐LIHC [the Cancer Genome Atlas Liver Hepatocellular Carcinoma]). Normalized microarray data matrix files obtained using the Affymetrix and Illumina platforms were directly downloaded. GSE14520 comprises 225 hepatocellular carcinoma tissues and 220 normal liver tissues. GSE76427 comprises 115 hepatocellular carcinoma tumor tissues and 52 adjacent non‐tumor tissues. We sourced the RNA‐seq transcriptome data, represented in fragments per kilobase of transcript per million mapped reads (FPKM) metrics, directly from the TCGA repository. This dataset encompassed clinicopathological data from 374 HCC specimens and 50 adjacent normal liver samples. Additionally, comprehensive genomic alteration data (capturing both somatic mutations and copy number variants (CNVs)) pertinent to HCC patients were procured for an in‐depth analysis. The R‐based tools, “maftools” and “Rcircos,” facilitated the identification of somatic mutations and CNVs within the TCGA‐LIHC cohort. For the validation set, RNA‐seq datasets accompanied by survival metrics were directly acquired from the International Cancer Genome Consortium (ICGC) portal (https://dcc.icgc.org/projects/LIRI‐JP), which included 202 control liver samples and 243 HCC specimens. As previously reported, the transcripts per kilobase million (TPM) values were considered equivalent to microarray data [15, 16]. We used R to transform the FPKM values obtained from the TCGA‐LIHC dataset into TPM values. Previously published data [17] revealed that batch effects can be minimized using the sva package “ComBat” algorithm to correct non‐biological technical biases in RNA‐seq and microarray data. Therefore, we utilized the R “sva” package to integrate the three datasets for further analysis.

The GSE112271 dataset encompassed single‐cell RNA sequencing details of seven HCC specimens, derived via the 10X Genomics platform. To meticulously scrutinize the scRNA‐seq dataset, a stepwise analytical framework was invoked. As a starting point, data preprocessing was accomplished through the Seurat toolkit. This stage paved the way for a comprehensive series of analyses. Utilizing the PercentageFeatureSet function, we delineated the proportion of mitochondrial gene presence. Additionally, we performed correlational evaluations to probe relationships among sequencing depth, mitochondrial gene content, and overall intracellular transcriptomes.

To bolster the precision of our analysis, only genes expressed in a minimum of five cells were considered. Cells were stringently curated based on explicit benchmarks: gene expression counts lying between 300 and 5000, a mitochondrial constitution below 10%, and an essential UMI threshold of 1000 per cell. This rigorous curation was geared towards ensuring cellular data fidelity. Post normalization of the scRNA‐seq data via the LogNormalize method, the curated dataset was primed for precise downstream analysis and interpretation.

Additionally, we acquired five HCC samples and five corresponding normal tissue specimens from the First Affiliated Hospital of Sun Yat‐sen University. Ethical approval for this investigative pursuit was conferred by the Institutional Review Board of the aforementioned institution.

### 2.2 Detection of m7G‐Related Genes and Identification of Molecular Subtypes

Genes associated with m7G were curated from the Gene Set Enrichment Analysis (GSEA) database (https://www.gsea‐msigdb.org/gsea/index.jsp) using the “7‐Methylguanosin” descriptor. A total of 29 genes pertaining to m7G were identified (see Table S1). From the amalgamated datasets (GSE14520, GSE76427, and TCGA‐LIHC), 17 genes with m7G association were singled out. Leveraging the expression profiles of these 17 genes, unsupervised stratification was executed utilizing the “consensusClusterPlus” R toolkit. During this stratification process, the cluster count, denoted as *k*‐values, ranged from 2 to 9. The optimal cluster count was pinpointed through a consensus clustering methodology. A visual representation of this coherent stratification, alongside pertinent clinical data, was constructed via the “pheatmap” R module.

### 2.3 Gene Set Variation Analysis

We employed the “GSVA” package in R to execute a gene set variation analysis, delving into the biological functionalities and potential pathways linked to m7G modification patterns. Prior to this analysis, the gene set designated as “c2.cp.kegg.v7.5.1.symbols” was extracted directly from the MSigDB repository.

### 2.4 Evaluation of TME Cell Infiltration by Single‐Sample Gene Set Enrichment Analysis

Utilizing the “GSVA” R toolkit, we conducted a single‐sample gene set enrichment analysis (ssGSEA) to gauge the infiltration rates of various immune cell populations. The ssGSEA technique interrogates individual oncological specimens based on gene profiles characteristic of specific immune cells. Adopting a deconvolution methodology, we assessed the presence of 24 distinct immune cell types, which encompassed activated B cells, CD4+ and CD8+ T cells in their activated states, dendritic cells (both activated and immature forms), natural killer cells characterized by CD56 bright and CD56 dim phenotypes, eosinophils, gamma delta T cells, immature B cells, myeloid‐derived suppressor cells (MDSCs), macrophages, mast cells, monocytes, NK‐T cells, neutrophils, plasmacytoid dendritic cells, regulatory T cells, T follicular helper cells, and the T helper cell subsets—Th1, Th2, and Th17 [18].

### 2.5 Identification and Functional Enrichment Analysis of Differentially Expressed Genes Between m7G Modification Patterns

In our study, the consensus clustering algorithm detected three significant m7G modification subtypes across the HCC patients. We subsequently used the R package “limma” to screen for differentially expressed genes (DEGs) among the three distinct m7G modification patterns and discovered DEGs with an adjusted *p* < 0.001. The DEGs were then assessed using Gene Ontology (GO) and Kyoto Encyclopedia of Genes and Genomes (KEGG) enrichment analyses. The GO and KEGG analysis results were visualized using the R package “ClusterProfiler.” A univariate Cox regression analysis was conducted per DEG, and those associated with a favorable prognosis were isolated for further study.

### 2.6 Estimation of the m7G Gene Signature and Other Related Biological Processes

Principal component analysis (PCA) was used to construct the m7Gscore according to the following formula [19]: m7Gscore = ∑(PC1_*i*_ + PC2_*i*_), where *i* represents the m7G phenotype genes expression. In the integrative dataset, we derived the m7Gscore for HCC patients using the designated equation. The cut‐off for survival was determined via the “survival” package in R. Based on this score, patients were stratified into groups with elevated or diminished m7Gscores. Prognostic comparisons were undertaken between these cohorts utilizing the “Survminer” R toolkit. To ascertain differences in potential responsiveness to chemotherapeutic and targeted therapeutic agents like cisplatin and sorafenib between the cohorts, we deployed the Wilcoxon test. In our pursuit to understand immunotherapeutic response dynamics, we extracted the immunophenoscore (IPS) data from The Cancer Immunome Atlas. Notably, the IPS serves as a salient marker for gauging responsiveness to CTLA‐4 and PD‐1 inhibitors and sheds light on differential therapeutic responses when targeting CTLA‐4 and PD‐1. The IPS metrics for HCC patients were sourced from the Cancer Immunome Atlas platform (https://tcia.at/patients) [20].

### 2.7 Validation of the Protein and mRNA Levels of m7G‐Related Genes

The Human Protein Atlas (HPA) (https://www.proteinatlas.org) was utilized for m7G‐related gene protein analysis. Seventeen m7G‐related genes in the HPA database were explored, 15 of which (*METTL1*, *WDR4*, *DCP2*, *DCPS*, *NUDT11*, *NUDT3*, *NUDT4*, *CYFIP1*, *EIF4E*, *LARP1*, *NCBP1*, *NCBP2*, *EIF4A1*, *EIF4G3*, and *IFIT5*) were identified in normal and HCC tissues.

Total RNA was isolated from HCC specimens employing the RN002plus RNA‐Quick Purification Kit (ESscience, CHINA) as per the manufacturer's protocol. The total RNA was then reverse transcribed into cDNA using a high‐capacity reverse transcription kit (Tsingke Biotechnology Co Ltd). Subsequent RT‐qPCR assays were conducted on a Light Cycler 480II (Roche) leveraging SYBR Green reagent (Tsingke Biotechnology Co Ltd). Relative mRNA expression levels of the target genes were quantified using the 2‐ΔΔCT methodology, where the cycling threshold (Ct) for each gene was noted. The RT‐qPCR was executed in strict adherence to the reagent guidelines, with each assay replicated thrice. Primer sequences utilized for the RT‐qPCR can be referenced in Table S2.

### 2.8 Western Blotting and Immunohistochemical Staining Analyses

Tissue samples were lysed in ice‐cold RIPA buffer to extract total protein, followed by quantification using the bicinchoninic acid (BCA) protein assay (Thermo Scientific). Equal amounts of protein were resolved by 10% SDS‐PAGE and subsequently transferred onto polyvinylidene difluoride (PVDF) membranes (Millipore). To prevent nonspecific antibody binding, membranes were blocked with 5% bovine serum albumin (BSA) in TBST at room temperature. The membranes were then incubated with primary antibodies against METTL1 (ab271063, Abcam), WDR4 (ab169526, Abcam), and GAPDH (60004‐1‐Ig, Proteintech) as a loading control. After thorough washing, HRP‐conjugated secondary antibodies (1:2000, CST) were applied. Immunoreactive bands were detected using enhanced chemiluminescence (ECL) reagents.

For immunohistochemical (IHC) analysis, sections derived from human hepatocellular carcinoma and matched normal liver tissues were used. Post paraffin‐removal and ethanol rehydration, antigen retrieval was performed in TRIS‐EDTA buffer under high‐pressure conditions for 7 min. To quench endogenous peroxidase activity, sections were treated with 3% H_2_O_2_ for 15 min. They were subsequently blocked with goat serum for an hour. Thereafter, sections were probed with primary antibodies against METTL1, WDR4, CD206, and FOXP3.

### 2.9 Statistical Analysis

Data were processed using R (4.1.3) statistical software. Spearman and distance correlation analyses were used to determine the correlation coefficients between the expression of m7G‐related genes and the infiltration of immune cells in the TME. In the case of comparisons of three or more groups, one‐way analysis of variance (ANOVA) and Kruskal–Wallis tests were performed [21]. We determined the cut‐off point using the survminer R program based on the correlation between the m7Gscore and patient survival outcomes. We employed the “surv‐cutpoint” function to bifurcate the m7Gscore, aiming to mitigate potential batch effects. Based on the optimal log‐rank statistic, patients were categorized into groups with elevated or reduced m7Gscore. Survival probabilities were assessed using the Kaplan–Meier estimator, with the log‐rank test delineating the statistical differences between curves. Univariate Cox proportional hazard analysis was conducted to determine hazard ratios (HRs) of both m7G‐associated genes and those reflecting the m7G phenotype. The distribution of TCGA‐LIHC patients across elevated and reduced m7Gscore categories was visualized utilizing the waterfall function in the maftools package. We used the RCircos R program to visualize the copy number variation landscape of 29 genes related to m7G across 23 pairs of chromosomes [22]. Two‐sided *p* values were calculated, with *p* < 0.05 considered to represent significance.

## 3 Results

### 3.1 scRNA‐Seq Data Preprocessing and Screening the Subclusters

**FIGURE 1:**
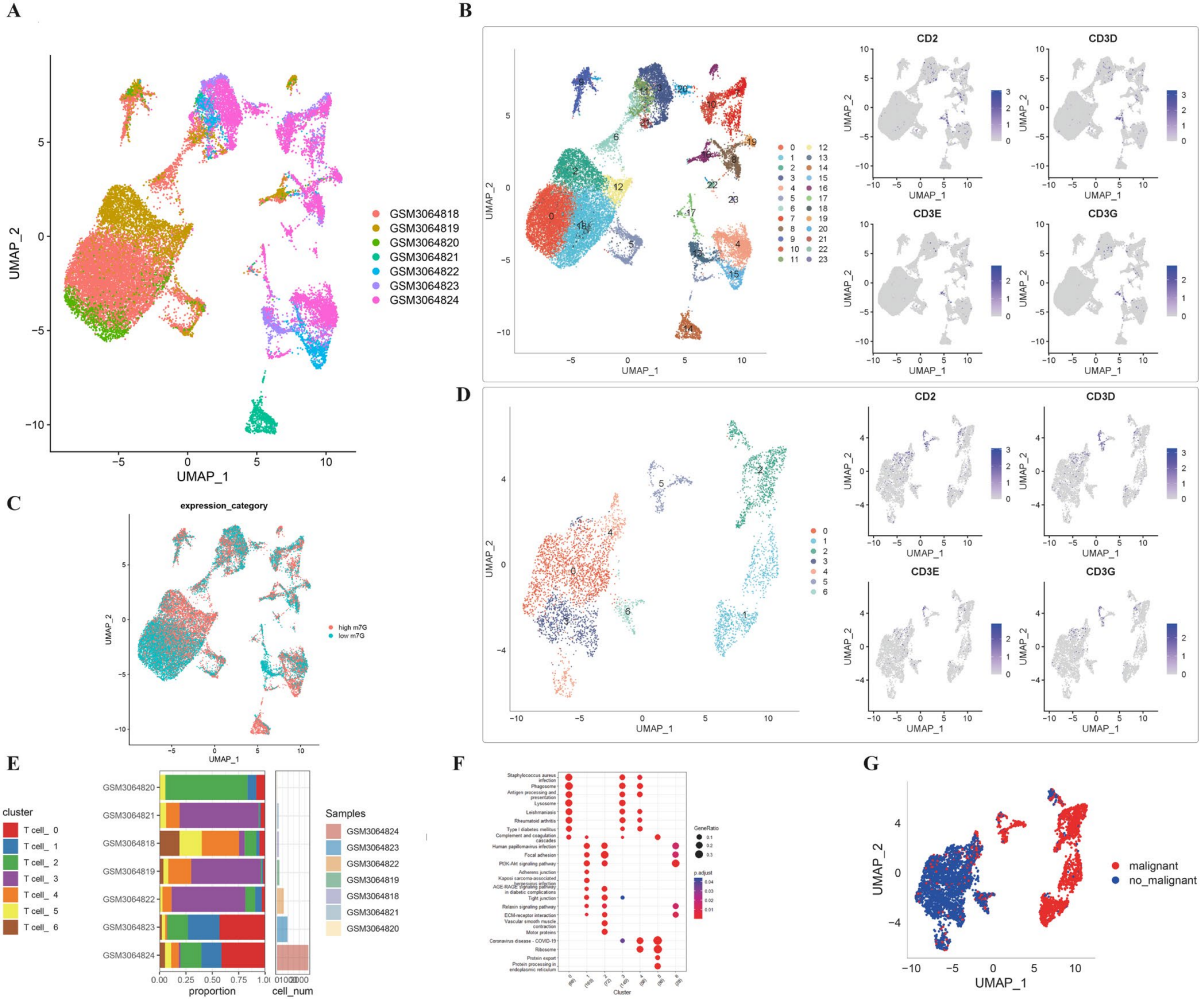
Characterization of T‐cell clusters using scRNA‐seq data from HCC patients. (A) UMAP visualization illustrating the distribution of 21 samples. (B) UMAP representation highlighting the distribution of 24 identified clusters, further distinguishing seven T‐cell subsets post‐clustering. (C) UMAP projection showing the distinction between high and low m7G gene expression levels. (D) UMAP representation distinguishing seven T‐cell subsets post‐clustering. (E) Detailed breakdown of subgroups within the cancerous tissue, complemented by the proportion and cell count in adjacent non‐cancerous tissue. (F) KEGG pathway enrichment analysis corresponding to the seven identified T‐cell subsets. (G) UMAP visualization depicting the spatial distribution of cells classified as malignant or non‐malignant based on predictions from the copykat package.

After log‐normalization and dimensionality reduction, 24 subpopulations were identified (Figures 1A,B and S1). Initially, we examined the distribution of 24 m7G‐related genes across the 24 clusters (refer to Figure S2). Recognizing the pivotal role that T cells play in the immune microenvironment of HCC, we selected four T cell marker genes: CD2, CD3D, CD3E, and CD3G. Within the 24 clusters, these T cell marker genes were predominantly expressed in clusters 11, 3, 21, 20, 7, 10, 17, 7, and 8 (Figure 1B).

Based on the m7G gene expression, clusters were classified into high and low m7G groups, depending on whether their average expression values were above or below the mean, respectively (Figure 1C). Based on the predominant T cell markers identified in the initial clustering, we conducted secondary clustering to further delineate the subpopulations of T cells, as illustrated in detail in Figure 1D. Further dimensionality reduction and clustering identified seven distinct T cell clusters used for subsequent analysis (Figure 1E,F). These clusters showed different proportions in each sample cohort, highlighting their heterogeneity (Figure 1E).

KEGG pathway analysis of differentially expressed genes (DEGs) within these clusters revealed significant involvement in pathways such as PI3K‐Akt signaling, focal adhesion, ECM‐receptor interaction, and adherens junctions (Figure 1F). Additionally, copy number variation (CNV) profiles distinguished neoplastic from non‐neoplastic cells within these clusters (Figure 1G).

### 3.2 m7G Genes Related to Hepatocellular Carcinoma Genetic Variation and Progression

Gene set enrichment analysis (GSEA) identified 29 m7G‐associated genes, of which 17 were selected from integrated datasets. Key genes such as CYFIP1, DCP2, EIF4E, EIF4E2, EIF4G3, LARP1, LSM1, METTL1, NCBP1, NCBP2, NUDT3, NUDT11, and WDR4 were found to be significant predictors of overall survival in HCC patients (Figure S3).

**FIGURE 2:**
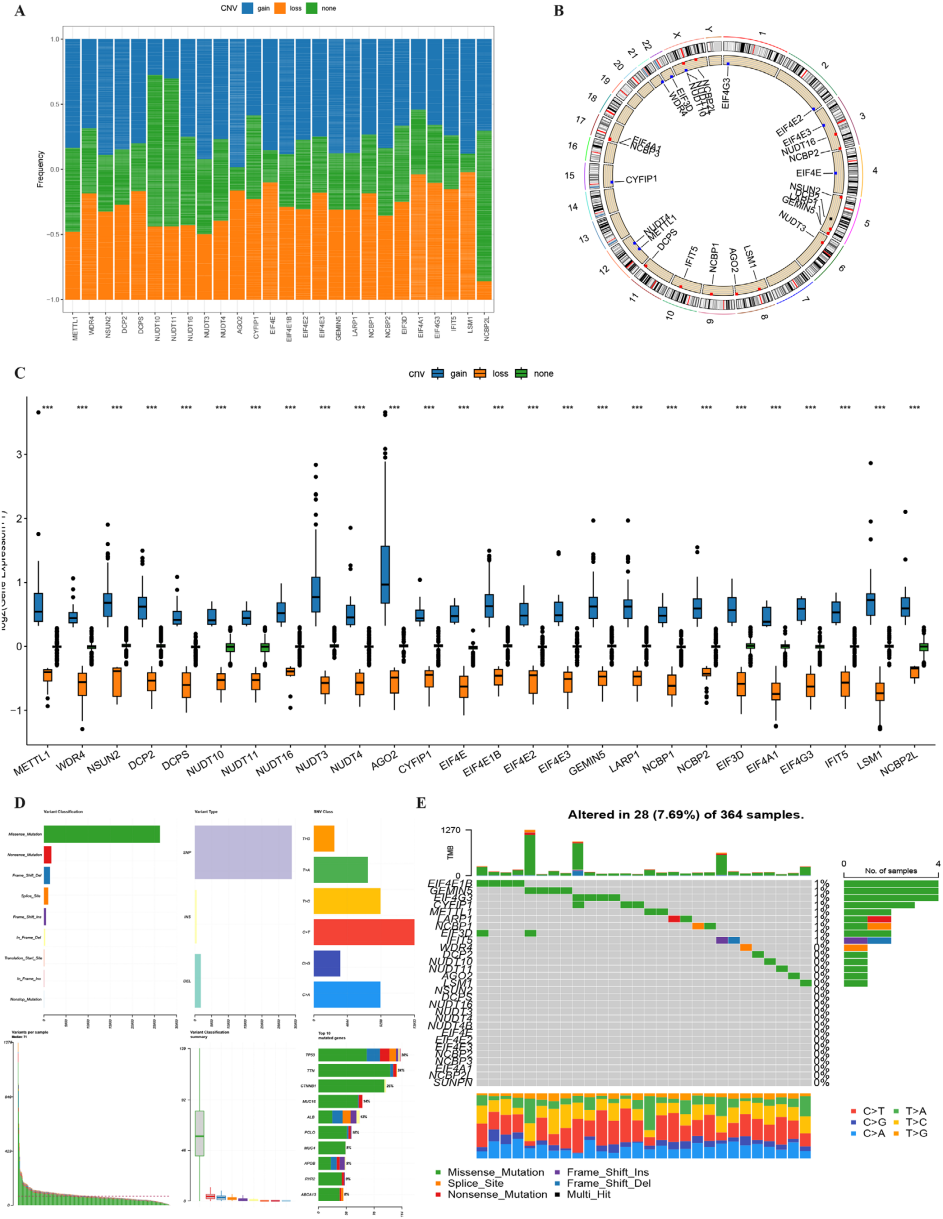
Overview of genetic alterations associated with m7G‐related genes in hepatocellular carcinoma (HCC). (A) Frequency of copy number variations (CNVs) observed in 29 m7G‐related genes within the TCGA‐LIHC cohort. Each column illustrates the respective alteration frequency. (B) Chromosomal localization of CNV alterations for m7G‐related genes. (C) Distribution of three distinct CNV types among the 29 m7G‐related genes. (D, E) Somatic mutation landscape and waterfall chart representation for m7G‐related genes in the TCGA hepatocellular carcinoma cohort. Out of the 364 HCC patients, 28 exhibited genetic alterations in the 29 m7G‐related genes, resulting in an alteration frequency of 7.69%. The numbers on the right display the mutation frequency for each respective gene, while individual columns represent distinct patients.

Analysis of CNVs revealed that genes like AGO2, NCBP2, EIF4E1B, GEMIN5, LARP1, NSUN2, METTL1, NUDT3, LSM1, EIF3D, NCBP2L, NUDT11, and NUDT10 showed amplification, whereas EIF4E2, CYFIP1, EIF4G3, DCP2, IFIT5, EIF4E, NCBP1, DCPS, NCBP3, EIF4E3, and EIF4A1 were prone to deletion (Figure 2A). The chromosomal locations of these CNVs are detailed in Figure 2B.

A comprehensive analysis of somatic mutations and CNVs from the TCGA‐LIHC dataset showed that 7.69% of samples had genetic alterations linked to m7G‐related genes (Figure 2C–E). This underscores the distinct genomic and transcriptomic characteristics of m7G‐related genes in HCC compared to healthy samples.

**FIGURE 3:**
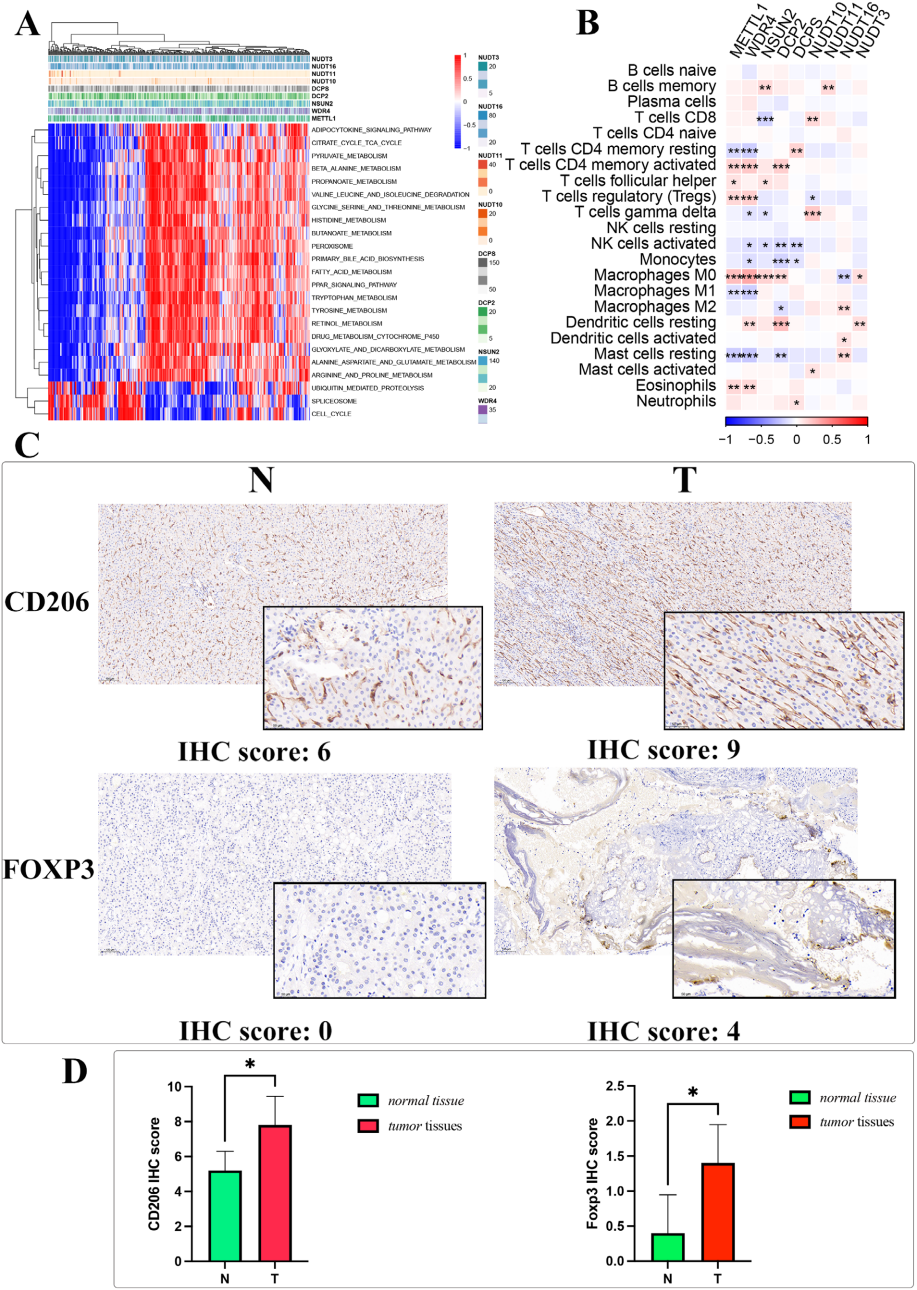
Characterization of m7G‐related genes' involvement. (A) Heatmap illustrating gene‐pathway correlations. (B) Correlation analysis of m7G‐related genes with immune cell infiltration. (C) IHC scoring in both tumor and normal tissues for macrophages and Treg cells. (D) Differential IHC staining scores in tumor versus normal tissues for CD206 and FOXP3. “IHC” refers to immunohistochemistry, and “Treg” to regulatory T cells. *p < 0.05.

Pathway analysis identified significant associations between m7G‐affiliated genes and pathways such as cell cycle regulation, fatty acid metabolism, and carbohydrate oxidation (Figure 3B). Notably, METTL1 and WDR4 were positively correlated with activated CD4 memory T cells, regulatory T cells, M0 macrophages, and eosinophils (Figure 3A). Immunohistochemistry (IHC) further confirmed the presence of macrophages and Tregs in HCC (Figure 3C,D), suggesting that m7G‐associated genes might influence HCC progression through immune modulation.

### 3.3 Characteristics of TME Cell Infiltration in Significant m7G Subtypes

**FIGURE 4:**
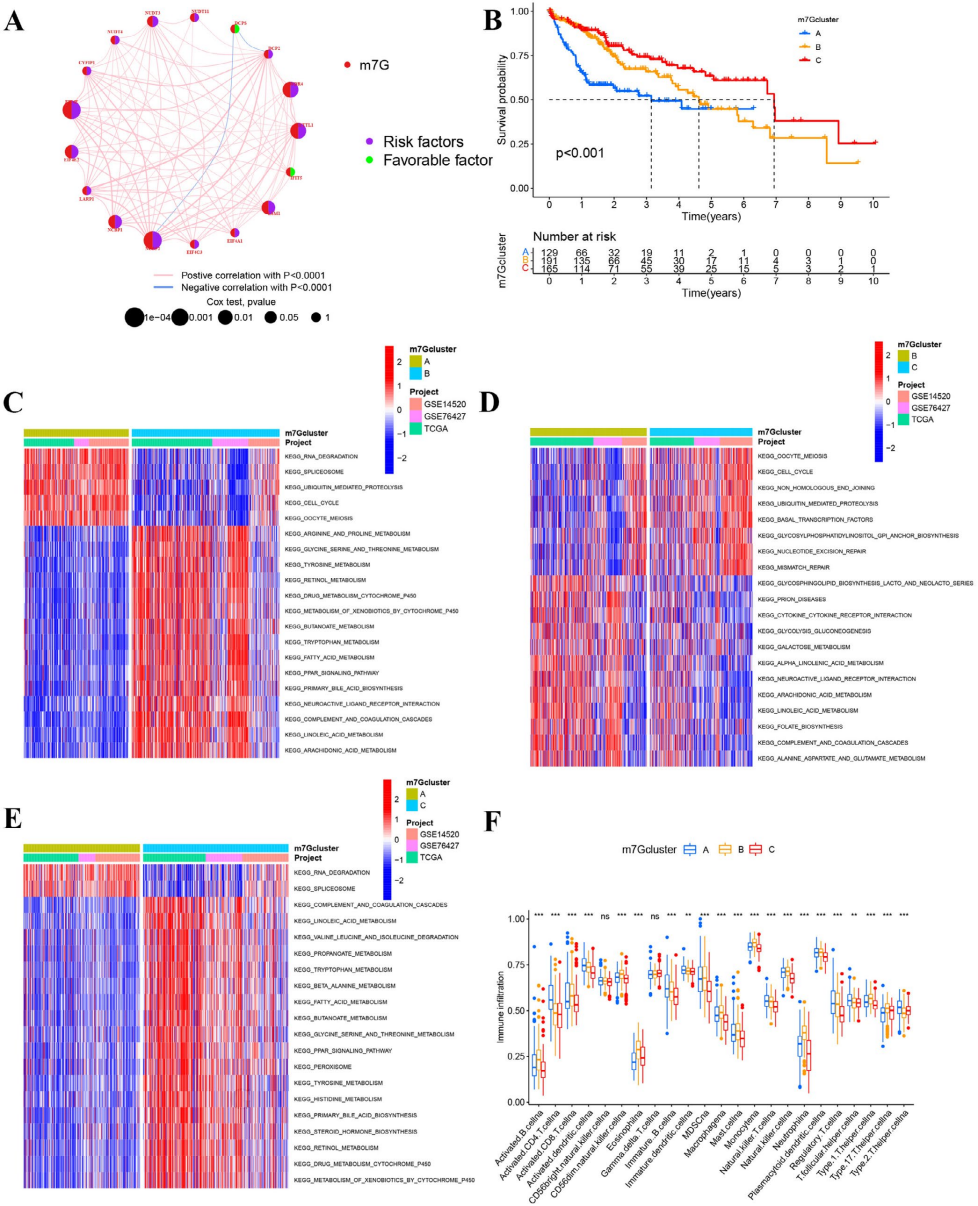
Characteristics of TME cell infiltration within prominent m7G subtypes. (A) Network diagram illustrating interactions among 17 m7G‐related genes in HCC. Lines between m7G‐related genes indicate their mutual interactions. Circle size correlates with the prognostic significance of each regulator, scaled by the *p*‐value. Purple dots within the circles signify risk factors for prognosis, while green dots indicate protective factors. (B) Kaplan–Meier survival curves representing overall survival (OS) for 485 HCC patients across an integrated cohort segregated by m7G clusters. Patient distribution among m7Gcluster A, m7Gcluster B, and m7Gcluster C was 129, 191, and 165, respectively (analyzed using the log‐rank test). GSVA enrichment analysis highlights the activation status of biological pathways among different m7G modification patterns. Red indicates activated pathways, while blue denotes inhibited pathways. (C) Comparative analysis between m7Gcluster A and m7Gcluster B. (D) Comparative analysis between m7Gcluster B and m7Gcluster C. (E) Comparative analysis between m7Gcluster A and m7Gcluster C. (F) Variation in immune cell infiltration across distinct m7G methylation modification patterns (**p < 0.01; ***p < 0.001).

A univariate Cox regression model was employed to elucidate the prognostic significance of the 17 m7G‐associated genes among HCC patients within a consolidated cohort. Interaction network diagrams were generated for these genes to visually represent their interconnections and prognostic implications (Figure 4A,B). The results underscored the prognostic relevance of m7G‐associated genes, shedding light on their clinical and biological pertinence.

Using the ConsensusClusterPlus package in R, patient cohorts were stratified based on divergent m7G modification patterns, which were derived from the expression metrics of the m7G‐associated genes (Figure S4A,B). This classification yielded three discernible modification patterns: Pattern A (129 instances), Pattern B (191 instances), and Pattern C (165 instances). A principal component analysis (PCA) highlighted distinct transcriptional demarcations across these three m7G clusters (Figure S4C).

The heatmap visualized associations between m7G‐associated genes and variables such as tumor stage, gender, age, research project, and m7G cluster designation (Figure S4D). Prognostically, cluster C emerged as the most favorable, while cluster A exhibited the least favorable outcomes (Figure 4B). A gene set variation analysis (GSVA) was conducted to discern the biological ramifications and enrichment of these disparate m7G modification profiles. As illustrated in Figure 4C, cluster A predominantly featured enrichments in genetic modifications and cellular pathways, encompassing RNA degradation, cell cycle regulation, and oocyte meiosis (Figure 4E). Cluster B was typified by metabolic pathways like arginine and proline metabolism, glycine, serine, threonine metabolism, fatty acid metabolism, xenobiotic metabolism by cytochrome P450, and primary bile acid biosynthesis (Figure 4C,D). In contrast, cluster C exhibited a blend of gene modifications and metabolic pathways (Figure 4D,E).

Furthermore, a single‐sample gene set enrichment analysis (ssGSEA) was conducted to appraise the variance in immune cell infiltration across the modification patterns. Intriguingly, samples from cluster B manifested heightened infiltration of innate immune cells, including natural killer cells, eosinophils, mature B cells, myeloid‐derived suppressor cells (MDSCs), macrophages, mast cells, and monocytes (Figure 4F). Yet, survival outcomes did not reflect a corresponding advantage for patients within this m7G cluster. Employing the ESTIMATE algorithm, the global immune and stromal cell infiltration landscapes across the three modification patterns were evaluated (Figure S4E,F). Notably, cluster B exhibited the apex stromal score, whereas clusters A and C bore diminished immune scores. This suggests a preponderance of non‐tumoral elements, such as immune and stromal cells, enveloping the cluster B subtype.

### 3.4 Generation of m7G Gene Signature and Correlation With Clinical Prognosis of HCC Patients

Further analysis of the three m7G modification patterns in HCC revealed 1304 differentially expressed genes (DEGs) associated with the m7G phenotype. Subsequent gene ontology (GO) and Kyoto Encyclopedia of Genes and Genomes (KEGG) enrichment analyses were executed on these DEGs (Figure S5A,B). GO analysis pinpointed biological processes, including DNA replication, chromosome segregation, chromosome organization, catalytic activity targeting DNA, and transcription co‐regulatory functions. Meanwhile, KEGG analysis emphasized gene associations with cellular processes such as the cell cycle, DNA replication, p53 signaling cascade, nucleocytoplasmic shuttling, and ubiquitin‐driven proteolysis. Utilizing a univariate Cox regression approach, 1032 genes were discerned to hold substantial prognostic relevance.

**FIGURE 5:**
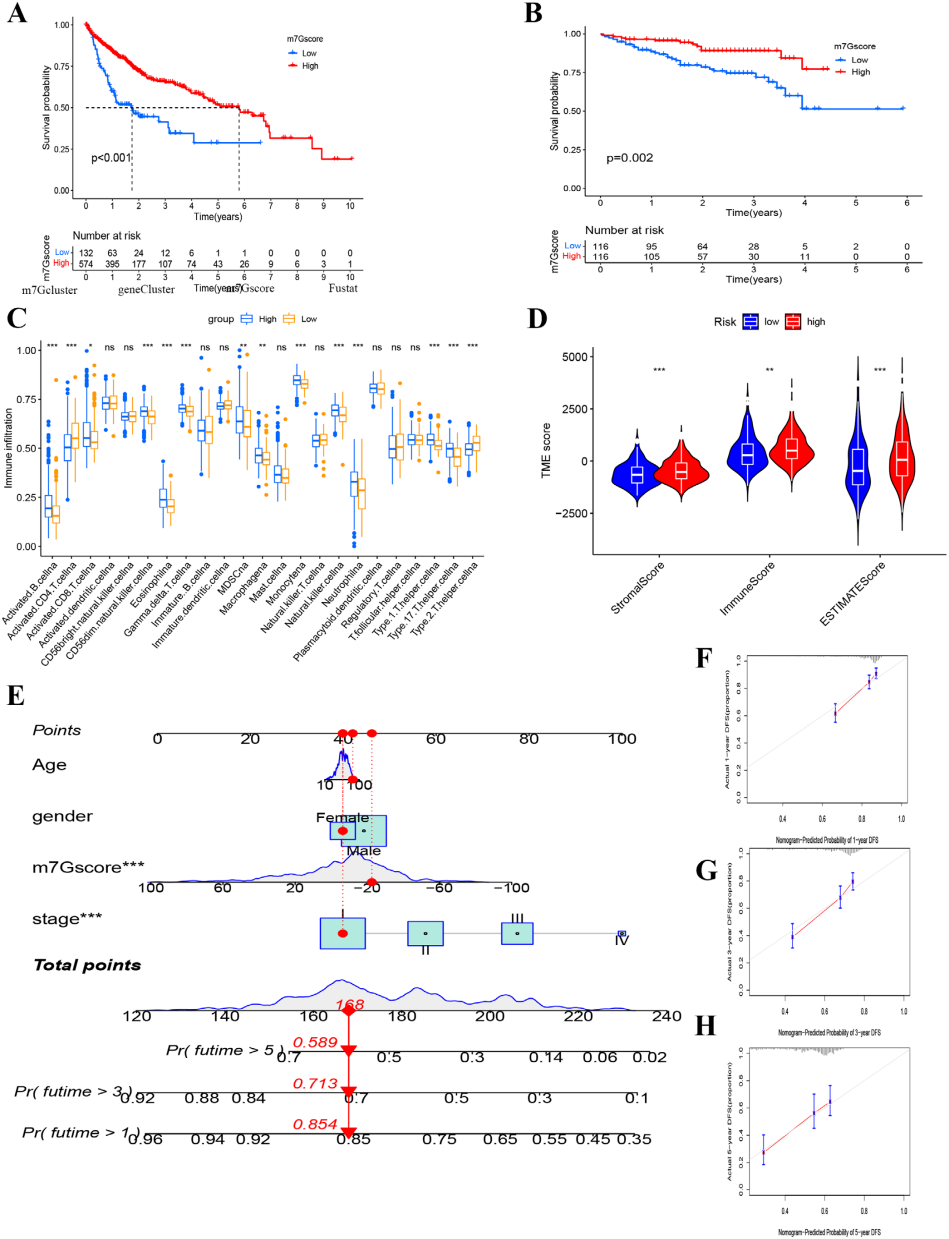
Elaboration of m7G signatures and formulation of a nomogram integrating the m7Gscore with clinicopathological features in HCC patients. (A) Kaplan–Meier survival curves for HCC patients with low and high m7Gscores in the training cohort. (B) Kaplan–Meier survival curves for HCC patients with low and high m7Gscores in the validation cohort (ICGC). (C) Disparities in immune cell infiltration among high and low m7Gscore subpopulations. (D) Assessment of the tumor microenvironment (TME) differences between the two m7Gscore groups. Associations between m7Gscore and both immune and stromal scores are shown. (E) A predictive nomogram for 1‐, 3‐, and 5‐year overall survival (OS) outcomes in combined cohorts. (F–H) Calibration plots validating the nomogram's accuracy for predicting 1‐, 3‐, and 5‐year OS outcomes. (*p < 0.05; **p < 0.01; ***p < 0.001; ns, not significant).

The aforementioned investigation solely factored in the collective patient cohort, thereby hampering precise predictions of m7G methylation modification patterns at the individual patient level. Recognizing individual heterogeneity and m7G modification intricacies, a scoring metric, termed “m7Gscore,” was devised, founded on the previously delineated 1032 phenotype‐centric genes. This m7Gscore provides a granular quantification of the m7G modification landscape in individual HCC patients. Subsequent assessments probed into the interplay between immune cell infiltration vis‐à‐vis the m7Gscore and its predictive prowess for survival trajectories. Employing the “survminer” R package, a threshold value of −27.2 was set, bifurcating patients into high‐ and low‐scoring cohorts. Survival analytics demonstrated enhanced survival prospects for high m7Gscore bearers (Figure 5A; *p* < 0.001). Analogously, the validation cohort underscored the inverse correlation of low m7Gscores with diminished survival prospects (Figure 5B; *p* = 0.002). Various clinical clusters persistently revealed favorable outcomes for the low m7Gscore cohort (Figure S6). Delving deeper into the immunological ramifications of the m7Gscore, a positive association emerged between CD4 memory resting T cells and the m7Gscore. Elevated risk scores resonated with heightened presence of immune constituents like activated B cells, activated CD8 T cells, CD56dim natural killer cells, eosinophils, gamma delta T cells, macrophages, monocytes, natural killer cells, and neutrophils (Figure 5C). As delineated in Figures 5D and S7A, the immune and stromal scores, in tandem with tumor purity, exhibited marked deviations across the risk‐based m7Gscore groups. Specifically, high‐risk cohorts manifested elevated TME stromal and immune scores relative to their low‐risk counterparts, whereas tumor purity predominated within the low‐risk group. Moreover, the m7G score and resting CD4 T memory cells (Figure S7B).

### 3.5 Establishing an Overall Survival Prediction Nomogram

Considering the potential clinical utility of the m7Gscore in prognosticating overall survival (OS) in HCC patients, a nomogram was formulated incorporating the m7Gscore alongside other pertinent clinicopathological features (Figure 5E). Insights gleaned from the nomogram underscored the importance of the m7Gscore as a noteworthy predictor of extended survival outcomes. Calibration curves revealed a commendable alignment between the predicted and actual survival rates based on the m7Gscore (Figure 5F–H). The area under the curve (AUC) values for 1‐, 3‐, and 5‐year survival using the m7Gscore stood at 0.677, 0.631, and 0.604, respectively (Figure S7C). Such metrics illuminate the m7Gscore's aptitude in forecasting 1‐year prognoses, though its predictive prowess diminishes slightly for 3‐ and 5‐year prognoses.

### 3.6 Correlation of m7Gscore With Genomic Instability and Anti‐HCC Therapies

**FIGURE 6:**
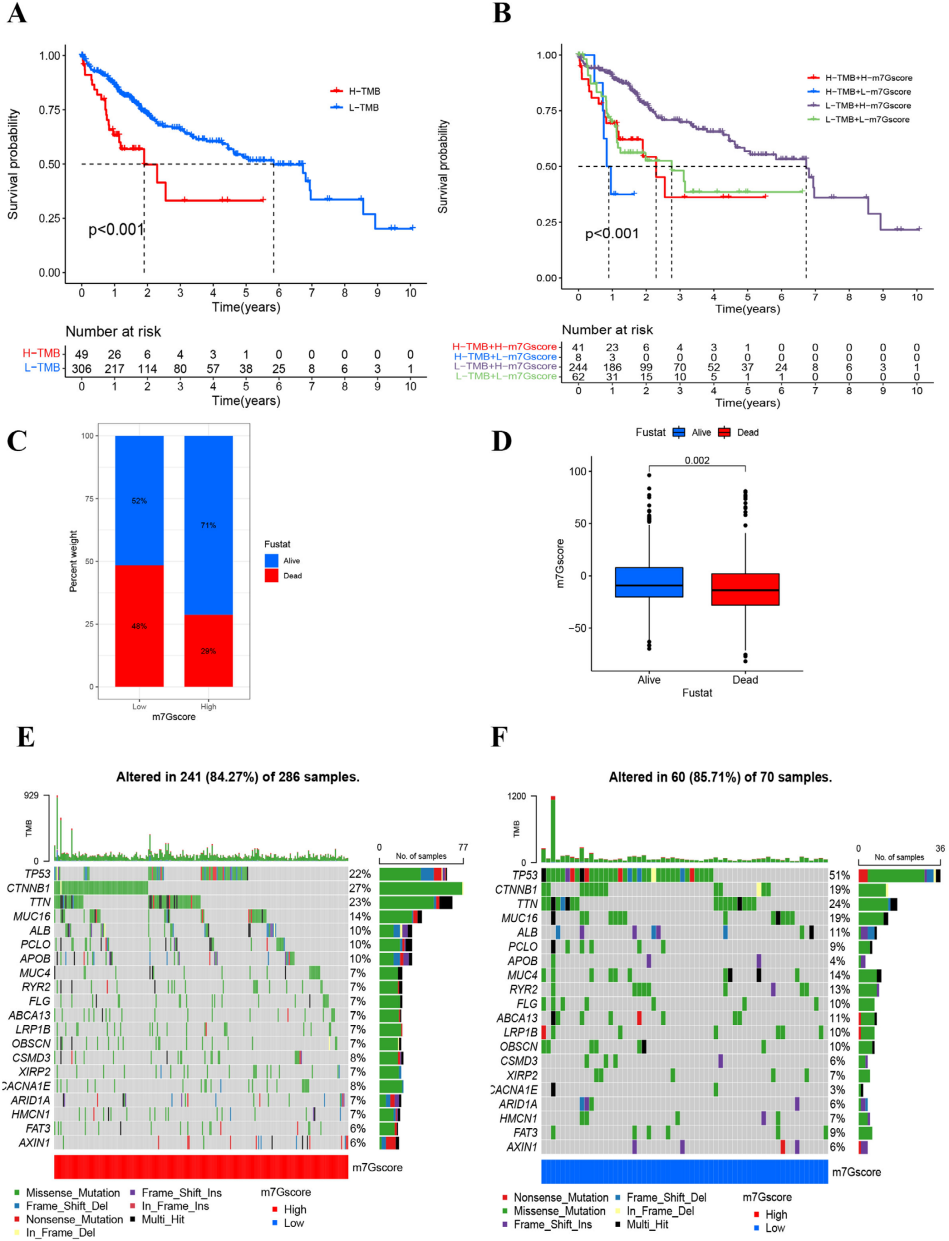
m7Gscore in relation to genomic instability. (A) Kaplan–Meier survival curves for patients categorized by low and high tumor mutation burden (TMB) using the Log‐rank test. (B) Survival evaluation of patient cohorts stratified by both m7Gscore and TMB through Kaplan–Meier plots. (C) Percentage distribution of patients with Fustat in either low or high m7Gscore categories. (D) Variation in the m7G‐scoring signature in relation to Fustat. Waterfall charts depicting somatic mutations in patients categorized by low and high m7Gscores. (E) Pattern of somatic mutations within the high m7Gscore cohort. (F) Distribution of somatic mutations within the low m7Gscore group.

Accumulating research posits that the tumor mutation burden (TMB) correlates with adverse outcomes in oncology (Figure 6A). Given this premise, our investigation honed in on discerning the relationship between the m7Gscore and TMB across distinct cohorts. Notably, elevated TMB was linked to deteriorating prognosis. A confluence of heightened TMB and diminished m7Gscore further intensified the correlation with unfavorable clinical trajectories (Figure 6B). Delving deeper into m7Gscore correlations, a significant association emerged between Fustat and m7Gscore (Figure 6C,D; *p* < 0.05). Employing the maftools package for analytical aid, a comparative analysis of somatic mutation frequencies between low and high m7Gscore cohorts revealed a pronounced mutation rate in the former. Within this spectrum, the TP53 gene emerged as the most recurrently mutated entity (Figure 6E,F).

**FIGURE 7:**
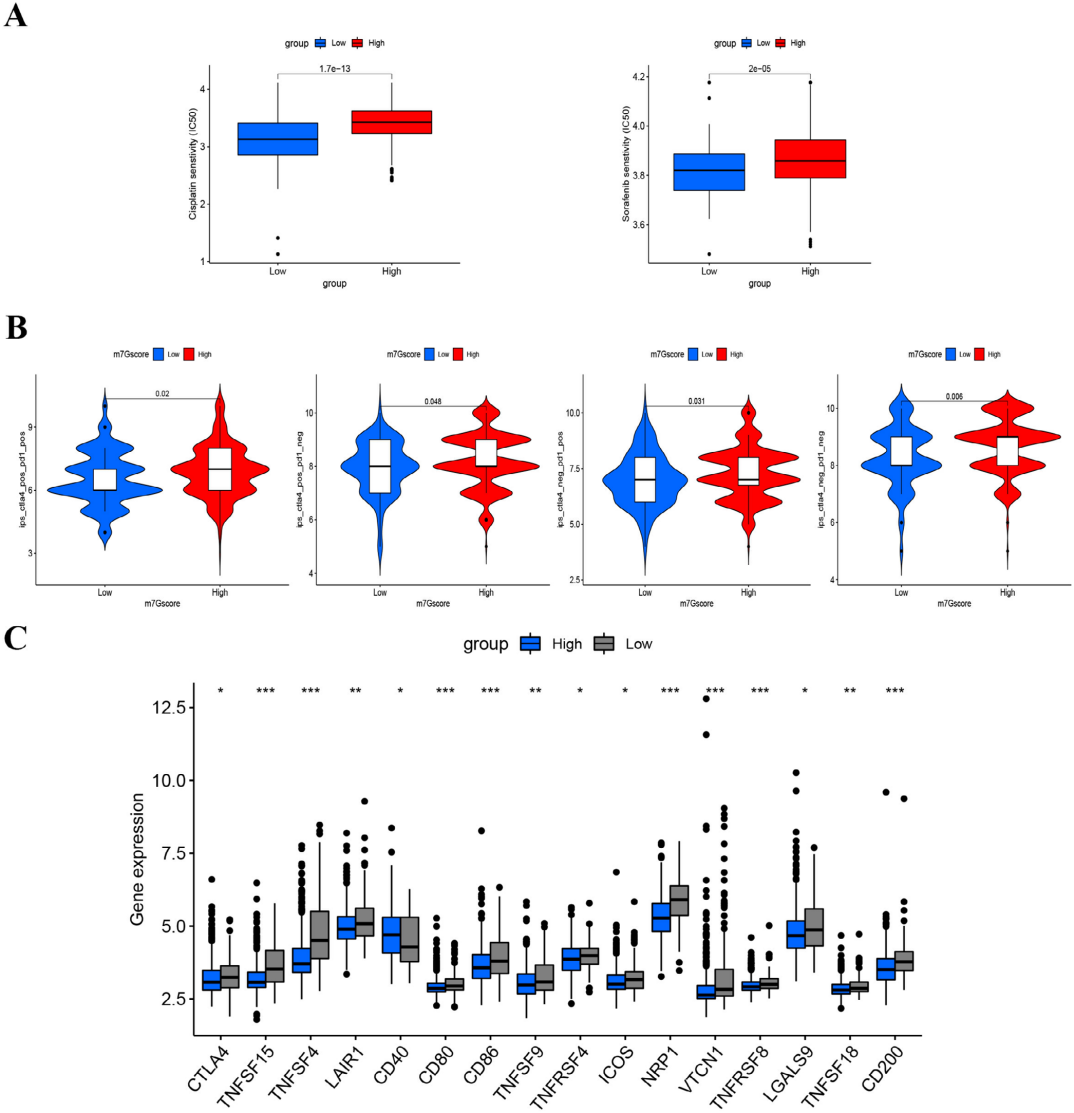
Implication of m7Gscore in HCC therapeutic approaches. (A) Box plots illustrating sensitivities of platinum‐based and targeted drugs, specifically cisplatin and sorafenib, in high and low m7Gscore cohorts. (B) Immunophenoscore distributions across different m7Gscore categories in patients presenting with various CTLA4 and PD‐1 statuses. (C) Differential expression of immune‐associated markers in the high versus low m7Gscore groups (*p < 0.05; **p < 0.01; ***p < 0.001; ns, not significant).

Considering the widespread use of platinum‐based and tyrosine kinase inhibitors as primary chemotherapy agents and targeted therapy in HCC treatment, we investigated whether the m7Gscore could predict responses to two of the first‐line chemotherapeutics and targeted therapy agents, cisplatin and sorafenib [23, 24]. Patients with high m7Gscores demonstrated considerable treatment sensitivity to both drugs, as shown in Figure 7A, implying that the m7Gscore contributes to predicting chemotherapy and targeted therapy responses.

To enhance the prediction of HCC patient responsiveness to immunotherapy, Immune Potential Scores (IPSs) of 317 patients were sourced from the TCIA database. Notably, antibodies targeting cytotoxic T lymphocyte antigen‐4 (CTLA‐4) and programmed cell death protein 1 (PD‐1) emerged as superior prognostic markers for HCC patient responses. Remarkably, each quadrant of patients with elevated m7Gscores registered higher IPSs. Moreover, these high m7Gscore patients demonstrated enhanced responsiveness to anti‐PD‐1 and anti‐CTLA‐4 treatments compared to their low m7Gscore counterparts (Figure 7B).

Additionally, an evaluation of various immune markers within the m7Gscore cohorts was undertaken, encompassing CTLA4, CD80, CD40, and VTCN1. Intriguingly, barring CD40, the other genes exhibited augmented expression levels within the low m7Gscore group (Figure 7C). This might elucidate the observed diminished survival rates in patients with a low m7Gscore. These insights underscore the potential of the m7Gscore as a robust biomarker for gauging patient responses to both chemotherapy and immunotherapy.

### 3.7 HPA Validation of m7G‐Related Genes

Proteomic analysis of m7G‐associated genes was undertaken utilizing the Human Protein Atlas (HPA) database. Due to the absence of immunohistochemical data in the HPA, two m7G‐related genes were omitted from the analysis. Consequently, immunohistochemical evaluations were performed for 15 m7G‐associated proteins. As delineated in Figure S8, proteins including EIF4E, EIF4G3, LARP1, METTL1, NCBP1, NUDT4, and NUDT11 displayed elevated expression in tumor specimens compared to their normal counterparts. Notably, DCPS manifested pronounced expression in both neoplastic and non‐neoplastic tissues, while DCP2, NCBP2, WDR4, and NUDT3 demonstrated intermediate expression across tumor and normal samples. Both malignant and benign specimens exhibited subdued expression of CYFIP1. Regrettably, immunostaining data for EIF4A1 and IFIT5 were unavailable in the HPA repository.

### 3.8 m7G‐Related mRNA Expression and Protein Level Validation

**FIGURE 8:**
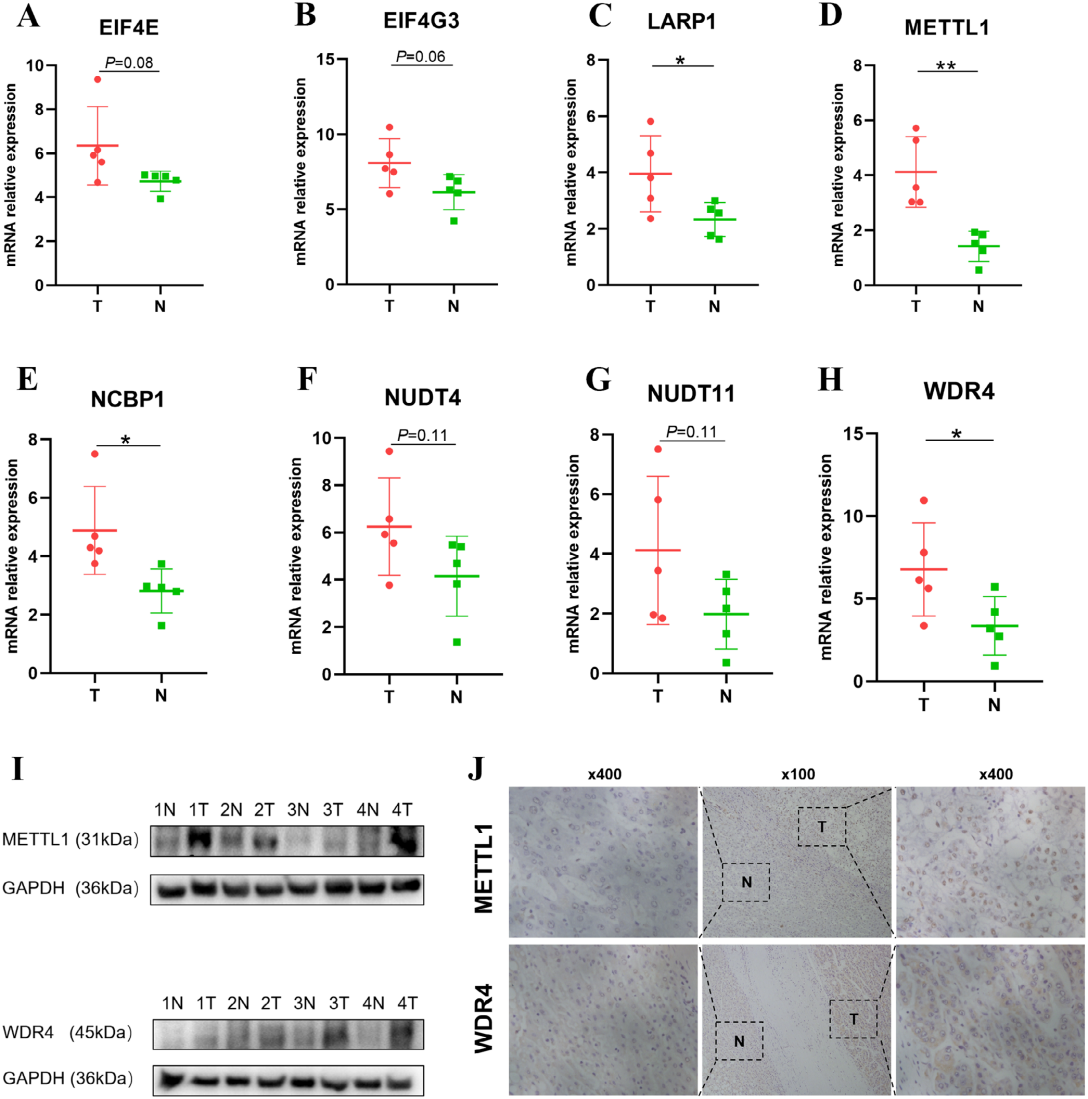
Expression profiling of m7G‐related genes in tumoral and adjacent normal specimens. (A–H) RT‐qPCR analyses revealing expression dynamics of EIF4E, EIF4G3, LARP1, METTL1, NCBP1, NUDT4, NUDT11, and WDR4. (I) Western blotting presents the expression levels of METTL1 and WDR4. (J) Immunohistochemistry (IHC) displays patterns in hepatocellular carcinoma versus paired normal tissues (*p < 0.05; **p < 0.01).

To corroborate our findings, we assessed the mRNA expression levels of m7G‐associated genes using real‐time quantitative polymerase chain reaction (RT‐qPCR). Notably, mRNA levels of LARP1, METTL1, WDR4, and NCBP1 were upregulated in tumor specimens (Figure 8A–H). Although EIF4E, EIF4G3, NUDT4, and NUDT11 expression levels did not exhibit a statistically significant differential between neoplastic and normal samples, their expression displayed an upward trend in hepatocellular carcinoma (HCC) tissues. Further, utilizing Western blot and immunohistochemistry (IHC) assays, we ascertained the protein abundance of METTL1 and WDR4. Our data revealed an upsurge in both METTL1 and WDR4 protein levels in HCC specimens (Figure 8I,J), which was in consonance with our prior bioinformatics insights.

## 4 Discussion

Genetic functions are regulated by RNA methylation changes [25], including N6‐methyladenosine (m6A), 5‐methylcytosine (m5C), N1‐methyladenosine (m1A), and m7G, among which m7G is the most prevalent at the 5′ caps of messenger RNA [26, 27, 28]. However, knowledge regarding the functions and regulatory mechanisms of m7G tRNA modifications in HCC development and progression remains limited.

Mounting studies have revealed that m7G alteration has a broad influence on tRNA, rRNA, and mRNA, and is required for various biological activities, including transcription elongation, pre‐mRNA splicing, and mRNA translation [4, 29, 30]. Research has shown that tRNA methylation and its associated catalytic enzymes METTL1 and WDR4 are enhanced in HCC and could negatively affect patient survival outcomes [6, 7]. Moreover, the tumor microenvironment could impact therapeutic responses and clinical outcomes [31]. It may be possible to identify potential prognostic features and immunotherapeutic strategies for HCC by investigating methylation patterns mediated by m7G‐related genes [12]. Identifying genetic variation and cancer heterogeneity would also further our understanding of RNA methylation and the discovery of RNA methylation‐based therapeutic targets [31].

One of the most revealing aspects of our investigation into hepatocellular carcinoma was undoubtedly the single‐cell sequencing insights. This state‐of‐the‐art approach allowed us to dissect HCC's cellular milieu, unmasking the intricate cellular heterogeneity that often remains concealed in bulk RNA sequencing.

Through rigorous data processing involving log‐normalization and dimensionality reduction, the single‐cell landscape unveiled several unique cellular subgroups within HCC. Each of these subpopulations represents a unique cellular identity with its own molecular signature, potentially pointing toward distinct roles within the tumor microenvironment.

Our focus on T‐cell populations, especially those expressing marker genes like CD2, CD3D, CD3E, and CD3G, has been particularly enlightening. T cells, being central players in tumor immunity, exhibit a spectrum of states in cancer—from activated effector cells mounting an attack on tumor cells to exhausted T cells that have been rendered ineffective by the tumor's immunosuppressive strategies. The expression patterns of these marker genes across different cellular clusters provided not only a snapshot of the T‐cell repertoire within HCC but also hinted at the dynamic interplay of immune activation and suppression within the tumor.

The role of m7G‐related genes in this cellular tapestry was another pivotal revelation. With their known functions in mRNA metabolism, their differential expression across cellular subpopulations potentially points toward nuanced roles in cellular fate decisions. For instance, the distinction of cellular clusters into high and low m7G expression categories may hint at differing metabolic states, proliferative capacities, or even susceptibilities to therapeutic interventions.

We commenced by investigating the clinical and biological significance of m7G‐associated genes. Subsequently, we employed consensus clustering to unveil distinct m7G subtypes based on the expression profiles of m7G‐related genes. It is noteworthy that unsupervised clustering processes often lack a definitive “gold standard” [32]. In our study, we identified a stable clustering solution with *K* = 3, substantiated by the consensus matrix and cumulative distribution function (CDF) analysis. Consequently, we stratified integrated HCC samples into three discrete methylation patterns predicated on the expression levels of 17 m7G‐related genes. Among these three m7G methylation modification patterns, m7Gcluster C exhibited the most favorable prognosis, while m7Gcluster A was associated with the least favorable outcomes.

Our analysis of TME cell infiltration in each m7G modification pattern revealed significant distinctions. m7Gcluster B exhibited hyperactivation of adaptive immunity, indicative of an immune‐inflamed phenotype, while m7Gcluster C displayed immune suppression, corresponding to an immune‐desert phenotype. m7Gcluster A showed a mixed immune response, including immune exclusion, where immune cells were mainly localized around tumor cells instead of infiltrating the parenchyma [33]. Non‐inflamed tumors fell into immune‐excluded or immune‐isolated phenotypes. Immune‐inflamed tumors, known as “hot tumors,” were characterized by immune cell infiltration into the tumor microenvironment [33, 34]. We hypothesized that immune‐inflamed cluster HCC patients would exhibit a favorable prognosis due to a potential immunotherapeutic response and low drug resistance. Surprisingly, patients in cluster C had the best prognosis in our survival analysis.

Using the GSVA method, we assessed the biological processes associated with the three m7Gclusters. m7Gcluster A was primarily linked to apoptosis, RNA degradation, and cell cycle regulation [35, 36], while m7Gcluster B was associated with tumor metabolism. Consequently, patients classified in m7Gcluster A were expected to have a poorer prognosis.

Our study identified 1304 DEGs associated with distinct m7G modification patterns based on GO and KEGG enrichment analyses. These results suggest that differences in mRNA transcriptomes in various m7G modification patterns may relate to cell cycle regulation, DNA replication, and p53 signaling pathways, which play a role in HCC oncogenesis and progression.

Furthermore, we conducted univariate Cox regression analysis, extracting 1032 genes associated with prognosis. To capture the overall m7G modification patterns in individual tumors, we introduced the concept of an individual patient's m7Gscore. Patients with low m7Gscores exhibited significantly worse prognoses, highlighting the m7Gscore as an independent prognostic biomarker for HCC. Higher m7Gscores correlated positively with antitumor immune cells, such as activated B cells, CD8+ T cells, natural killer T cells, macrophages, and neutrophils, suggesting improved overall survival outcomes and potential benefits from immunotherapy.

Despite these findings, the practical application of the m7G scoring signature in immunotherapy remains unexplored. Both training and validation of high m7Gscore groups demonstrated enhanced survival benefits, potentially attributed to increased immune infiltration and higher stromal and immune scores. Combining m7Gscore with TMB for predicting HCC patient survival and immunotherapeutic strategies revealed a significant survival advantage for patients with low TMB scores and high m7Gscores in HCC (*p* < 0.001). Conversely, patients in the low‐m7Gscore group with high TMB scores exhibited shorter survival times.

Somatic mutation analysis revealed that CTNNB1 mutations were most common (27%) in patients with high m7Gscores, while TP53 mutations were predominant (51%) in patients with low m7Gscores. TP53 mutations have been associated with a poor prognosis in HCC and hypoxia‐induced HCC stemness [37, 38], consistent with our findings that the low m7Gscore subgroup with a high TP53 mutation rate had an inferior prognosis compared to the high m7Gscore subgroup.

The therapeutic and prognostic landscape for HCC patients has seen notable enhancements through the application of ICI treatments, encompassing anti‐PD‐1 and CTLA‐4 therapies [39, 40]. A further analysis was conducted to determine the predictive value of the m7Gscore regarding anti‐PD‐1 and anti‐CTLA‐4 immunotherapy among HCC patients. It was concluded that individuals with high m7Gscores might benefit from immunotherapy targeting CTLA4/PD‐1 inhibitors.

Ultimately, we examined the expression profiles of m7G‐associated genes in HCC relative to normal liver tissues. An elevated protein expression of EIF4E, EIF4G3, LARP1, METTL1, NCBP1, NUDT4, and NUDT11 was discerned in the carcinoma samples, whereas the WDR4 expression was comparable between the tumor and adjacent non‐tumorous tissues. After determining that these eight m7G‐related genes were expressed in HPA, we utilized real‐time PCR to examine their mRNA expression levels. *LARP1*, *METTL1*, *WDR4*, and *NCBP1* expression levels were substantially upregulated. *EIF4E*, *EIF4G3*, *NUDT4*, and *NUDT11* were more likely to be expressed in HCC than in normal tissues. As described previously [6, 7], METTL1 and WDR4 are key factors in m7G tRNA modification; thus, we further examined the protein expression levels of METTL1 and WDR4 in HCC and corresponding normal tissues by western blotting and IHC. METTL1 and WDR4 protein levels were significantly higher in HCC, consistent with the results from previous studies using the same techniques [6, 7]. In general, the mRNA and protein expression level data aligned with the TCGA, ICGC, and GEO outcomes.

This study delved into the gene expression patterns linked to m7G in HCC, augmenting our understanding of their functional roles. The m7Gscore holds promise for identifying biomarkers, guiding treatment decisions for HCC patients, and serving as a practical tool for evaluating m7G‐methylation status, predicting clinical outcomes, characterizing TME infiltration, and assessing responses to chemotherapy, targeted therapies, and immunotherapies. However, our investigation does have certain limitations. Firstly, our bioinformatics analyses relied on publicly available datasets, constraining further exploration. While public datasets provide a valuable resource for large‐scale analysis, they are limited by retrospective study designs and potential batch effects. Additionally, clinical follow‐up data may vary in completeness. Prospective validation in independent cohorts would strengthen the clinical applicability of our findings. Secondly, the predictive utility of the m7Gscore and m7G‐related genes warrants validation in larger datasets and through additional clinical samples. Subsequent in vitro and in vivo experimental research is essential to validate the association between the m7Gscore and HCC.

## 5 Conclusion

In this study, insights from single‐cell sequencing unveiled the intricate cellular heterogeneity within HCC, often obscured by bulk RNA sequencing, enriching our understanding of unique cellular identities. We further illustrated how variations in m7G modification patterns significantly contribute to the complexity of individual tumor microenvironments. By analyzing m7G patterns and scores in individual HCC patients, we provide deeper insights into the tumor landscape, paving the way for more targeted therapeutic strategies.

## Author Contributions

**Jiahua Liang:** conceptualization (equal), data curation (equal), formal analysis (equal), writing – original draft (equal), writing – review and editing (equal). **Mingjian Ma:** validation (equal), writing – original draft (equal). **Borui Xu:** visualization (equal), writing – original draft (equal). **Cheng Zhong:** validation (equal), writing – review and editing (equal). **Guangyan Zeng:** writing – review and editing (equal). **Huijiao Lu:** investigation (equal), methodology (equal), resources (equal). **Jiaming Lai:** project administration (equal). **Jiancong Chen:** project administration (equal), validation (equal), writing – review and editing (equal).

## Ethics Statement

The study was also in accordance with the Helsinki Declaration and approved by the Ethics Committee of the First Affiliated Hospital of Sun Yat‐sen University [no. [2024]459]. Written informed consents were obtained from all patients.

## Conflicts of Interest

The authors declare no conflicts of interest.

## Supporting information


**FIGURE S1.** Single‐cell sequencing analysis of HCC specimens. (A) Correlational analysis encompassing nFeature vs. nCount, percent.mt versus nCount, and percent.mt versus nFeature. (B) Violin plots depicting the RNA characteristic count (nFeature RNA) and the absolute UMI count (nCount RNA) prior to cell quality control measures. (C) Violin plots showcasing the RNA characteristic count (nFeature RNA) and the absolute UMI count (nCount RNA) post quality control filtering. (D) Principal Component Analysis (PCA) executed on the single‐cell RNA sequencing data. (E) Analytical clustering tree with resolution parameter set to 1.2.

**FIGURE S2.** Single‐cell expression patterns of m7G‐related genes.

**FIGURE S3.** (A–M) Kaplan–Meier plots highlighting the prognostic implications of m7G‐associated gene expression in HCC patients from the TCGA‐LIHC cohort.

**FIGURE S4.** (A) Application of consistency clustering to produce a color‐coded consistency matrix heatmap for *k* = 3, with color gradient spanning from white (value: 0) to dark blue (value: 1). (B) Delta area curves from consistent clustering visualizing the relative shifts in the cumulative distribution function (CDF) curve area for each category of *k* relative to *k* − 1. (C) Principal component analysis plot delineating the transcriptomic variations among the three m7Gclusters. (D) Heatmap labeled with survival status (Fustat), age, gender, stage, and m7G methylation modification patterns (m7Gcluster). (E, F) Box plots indicating disparities in immune score and stromal score among the three m7Gclusters.

**FIGURE S5.** Enrichment analysis. (A) Bar plots illustrating the functional annotation of differentially expressed genes (DEGs) across varied m7Gclusters using GO (Gene Ontology) analysis. The color intensity of the bars indicates the number of enriched genes. (B) Functional annotation of the Differentially Expressed Genes (DEGs) across the different m7Gclusters using KEGG (Kyoto Encyclopedia of Genes and Genomes) pathway analysis.

**FIGURE S6.** Prognostic relevance of m7G‐scoring signature in distinct clinical cohorts. The Kaplan–Meier method paired with Log‐rank tests was employed. Analysis includes patients with (A) age > 65, (B) age ≤ 65, (C) female, (D) male, (E) Stage I + II, (F) Stage III + IV.

**FIGURE S7.** Correlational and predictive analyses based on m7Gscore. (A) Analysis showcasing the correlation between m7Gscore and TumorPurity. (B) The association between m7Gscore and immune cell infiltration. (C) ROC (receiver operating characteristic) curves illustrating the predictive capability for 1‐, 3‐, 5‐, and 10‐year survival based on m7Gscore.

**FIGURE S8.** Expression profiles of m7G‐associated proteins in tumoral and normal tissues according to the HPA (Human Protein Atlas) Database. (A) CYFIP1 expression (B) DCP2 expression (C) DCPS expression (D) EIF4A1 expression (E) EIF4E expression (F) EIF4G3 expression (G) IFIT5 expression (H) LARP1 expression (I) METTL1 expression (J) NCBP1 expression (K) NCBP2 expression (L) NUDT3 expression (M) NUDT4 expression (N) NUDT11 expression (O) WDR4 expression.

**TABLE S1.** Source of 29 m7G‐related genes.

**TABLE S2.** Forward primer and Reverse primer sequences for 8 m7G‐related genes.

**TABLE S3.** List of abbreviations used in the manuscript.

## Data Availability

Data generated or used in this study are available from the corresponding authors upon reasonable request.
