# Supplementary figures and images for: Integrated Analysis of Single‐Cell and Bulk RNA‐Sequencing Defines N7‐Methylguanosine (m7G)‐Mediated Modifications' Role in Prognosis and the Tumor Immune Microenvironment in Hepatocellular Carcinoma
Source: Cancer Med. 2025 Jun 9;14(11):e70992. doi: 10.1002/cam4.70992 (PMC12146900; doi:10.1002/cam4.70992)

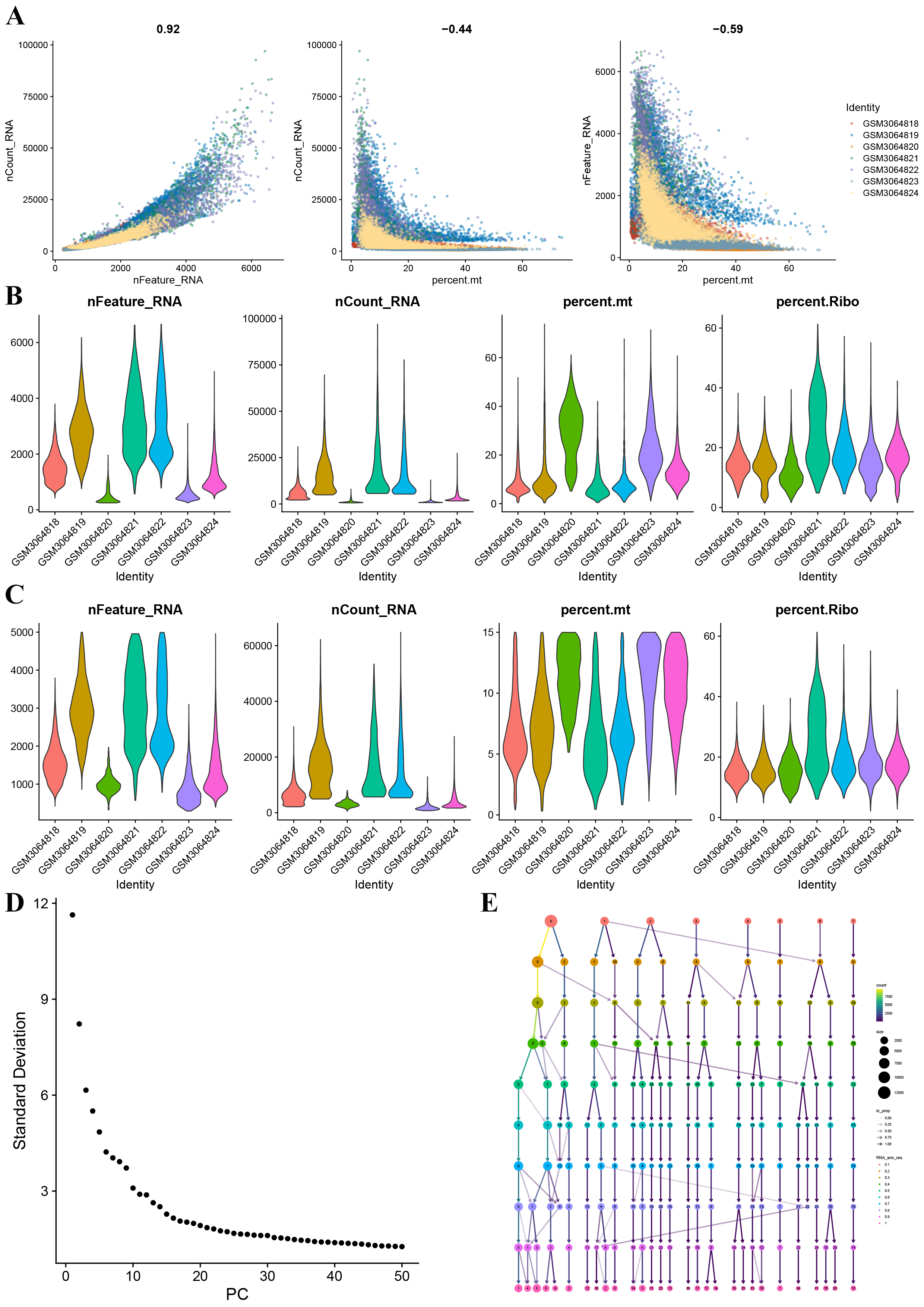

Supplement: Supplementary file 1 — FIGURE S1. Single‐cell sequencing analysis of HCC specimens. (A) Correlational analysis encompassing nFeature vs. nCount, percent.mt versus nCount, and percent.mt versus nFeature. (B) Violin plots depicting the RNA characteristic count (nFeature RNA) and the absolute UMI count (nCount RNA) prior to cell quality control measures. (C) Violin plots showcasing the RNA characteristic count (nFeature RNA) and the absolute UMI count (nCount RNA) post quality control filtering. (D) Principal Component Analysis (PCA) executed on the single‐cell RNA sequencing data. (E) Analytical clustering tree with resolution parameter set to 1.2. [file CAM4-14-e70992-s011.tif]

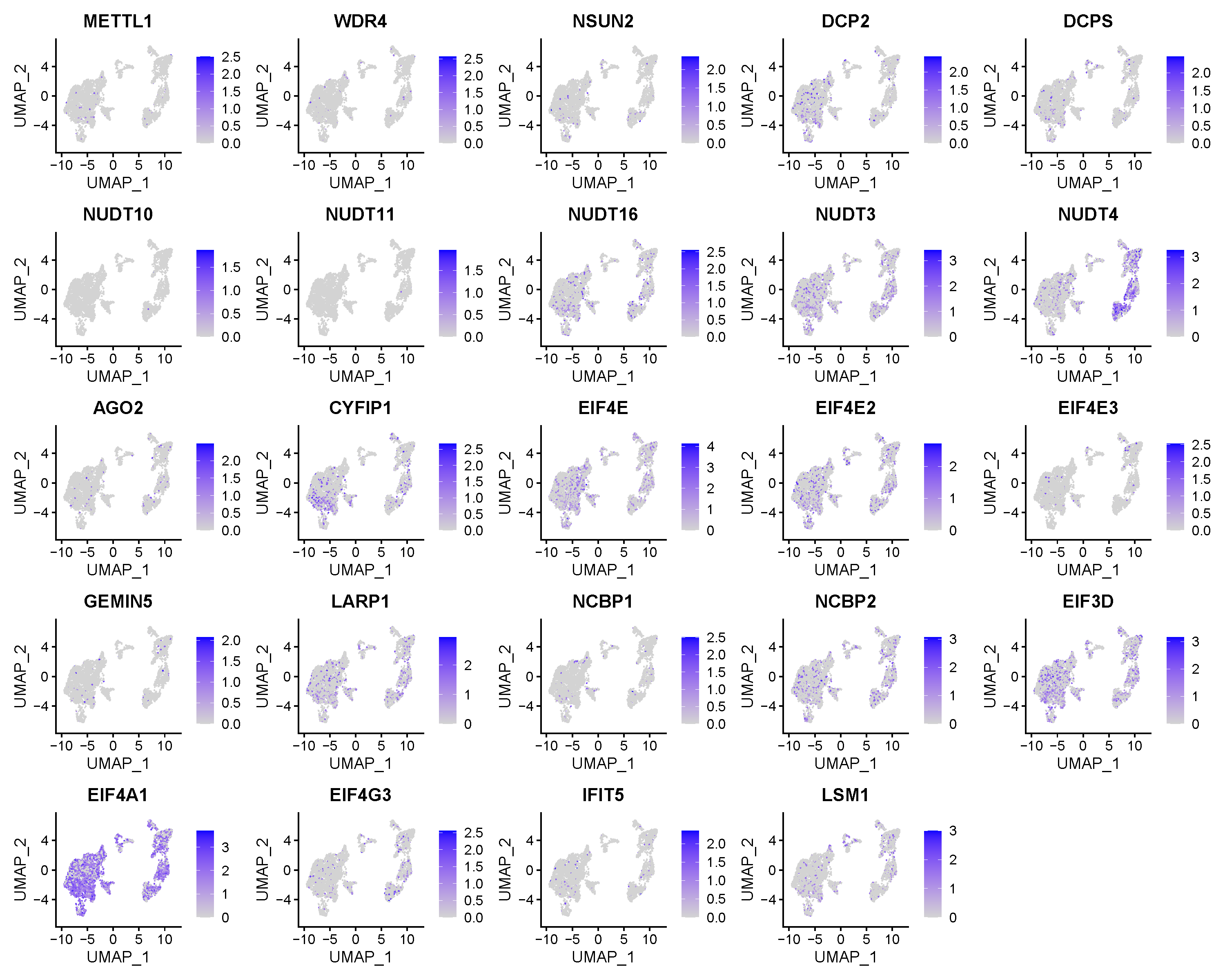

Supplement: Supplementary file 2 — FIGURE S2. Single‐cell expression patterns of m7G‐related genes. [file CAM4-14-e70992-s003.tif]

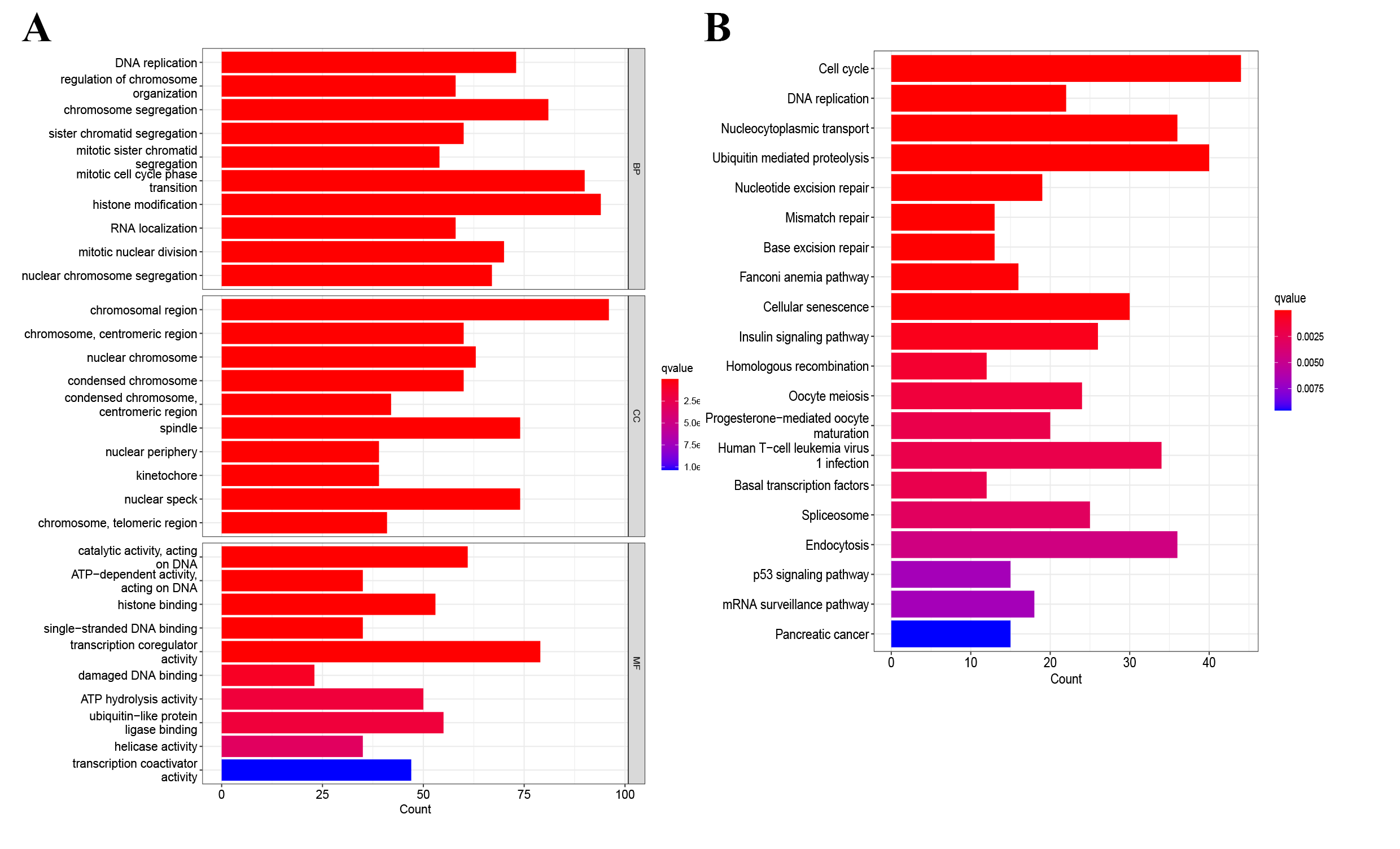

Supplement: Supplementary file 5 — FIGURE S5. Enrichment analysis. (A) Bar plots illustrating the functional annotation of differentially expressed genes (DEGs) across varied m7Gclusters using GO (Gene Ontology) analysis. The color intensity of the bars indicates the number of enriched genes. (B) Functional annotation of the Differentially Expressed Genes (DEGs) across the different m7Gclusters using KEGG (Kyoto Encyclopedia of Genes and Genomes) pathway analysis. [file CAM4-14-e70992-s007.tif]

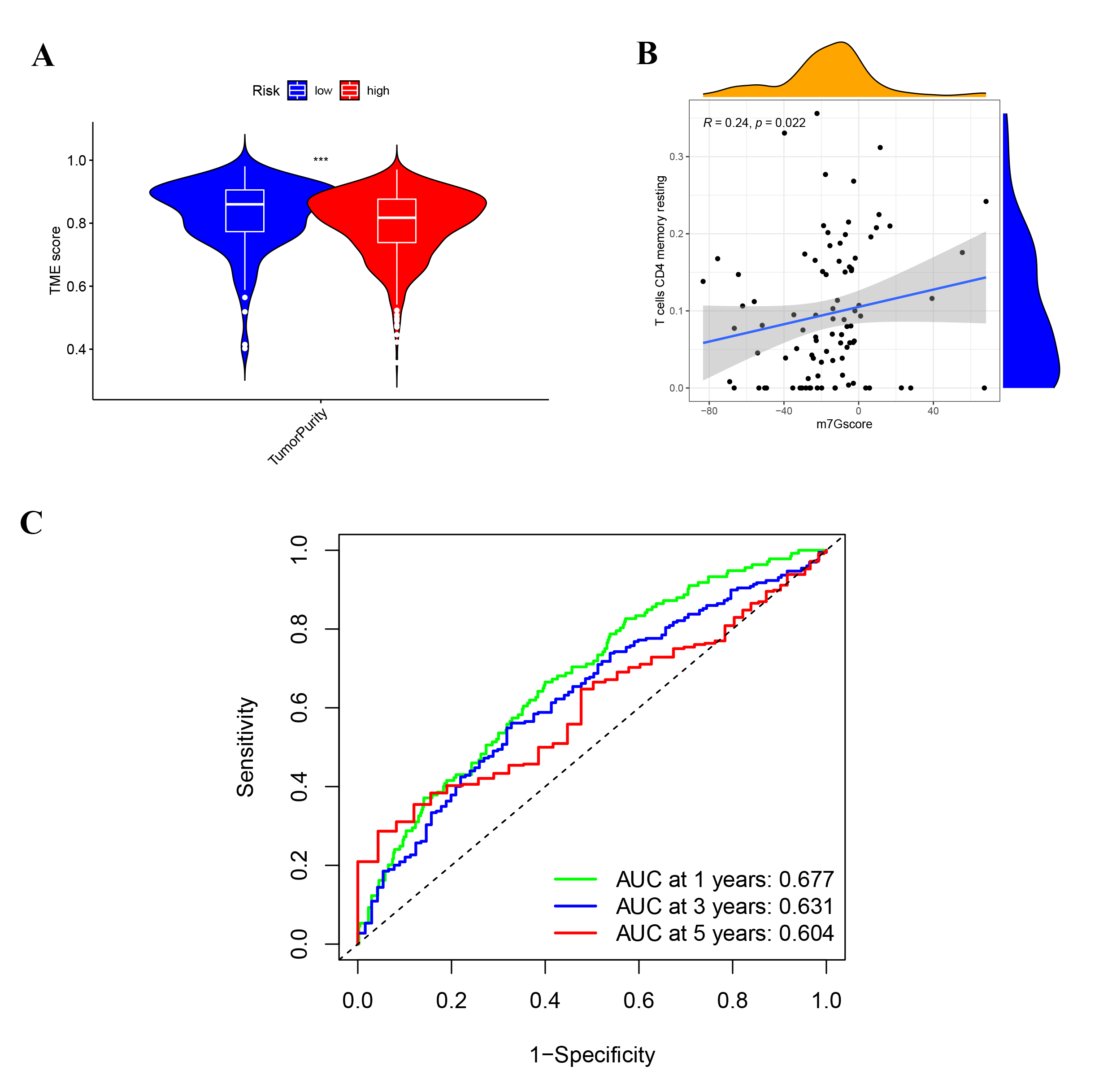

Supplement: Supplementary file 7 — FIGURE S7. Correlational and predictive analyses based on m7Gscore. (A) Analysis showcasing the correlation between m7Gscore and TumorPurity. (B) The association between m7Gscore and immune cell infiltration. (C) ROC (receiver operating characteristic) curves illustrating the predictive capability for 1‐, 3‐, 5‐, and 10‐year survival based on m7Gscore. [file CAM4-14-e70992-s006.tif]

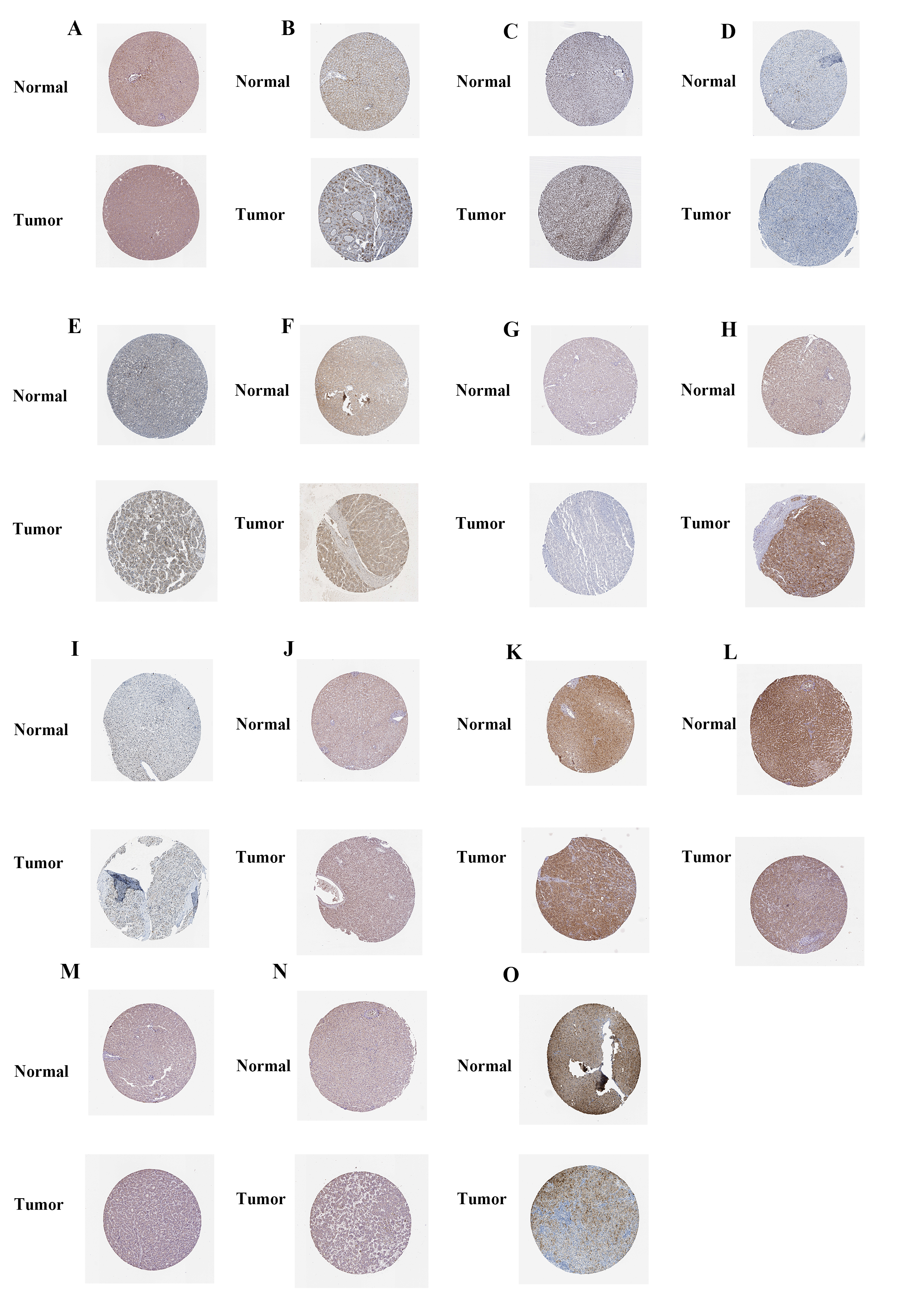

Supplement: Supplementary file 8 — FIGURE S8. Expression profiles of m7G‐associated proteins in tumoral and normal tissues according to the HPA (Human Protein Atlas) Database. (A) CYFIP1 expression (B) DCP2 expression (C) DCPS expression (D) EIF4A1 expression (E) EIF4E expression (F) EIF4G3 expression (G) IFIT5 expression (H) LARP1 expression (I) METTL1 expression (J) NCBP1 expression (K) NCBP2 expression (L) NUDT3 expression (M) NUDT4 expression (N) NUDT11 expression (O) WDR4 expression. [file CAM4-14-e70992-s002.tif]
