# Supplementary material for: Integrated Analysis of Single‐Cell and Bulk RNA‐Sequencing Defines N7‐Methylguanosine (m7G)‐Mediated Modifications' Role in Prognosis and the Tumor Immune Microenvironment in Hepatocellular Carcinoma
Source: Cancer Med. 2025 Jun 9;14(11):e70992. doi: 10.1002/cam4.70992 (PMC12146900; doi:10.1002/cam4.70992)
Supplement: Supplementary file 11 — TABLE S3. List of abbreviations used in the manuscript. [file CAM4-14-e70992-s005.docx]

## Supplementary Table S3. List of Abbreviations Used in the Manuscript

| Abbreviation | Full Term |
| --- | --- |
| HCC | Hepatocellular Carcinoma |
| m7G | N7-Methylguanosine |
| TME | Tumor Microenvironment |
| scRNA-seq | Single-Cell RNA Sequencing |
| RT-qPCR | Reverse Transcription Quantitative Polymerase Chain Reaction |
| WB | Western Blot |
| IHC | Immunohistochemistry |
| TCGA | The Cancer Genome Atlas |
| GEO | Gene Expression Omnibus |
| ICGC | International Cancer Genome Consortium |
| CNV | Copy Number Variation |
| DEG | Differentially Expressed Gene |
| GSVA | Gene Set Variation Analysis |
| ssGSEA | Single Sample Gene Set Enrichment Analysis |
| GO | Gene Ontology |
| KEGG | Kyoto Encyclopedia of Genes and Genomes |
| PCA | Principal Component Analysis |
| IPS | Immunophenoscore |
| ICI | Immune Checkpoint Inhibitor |
| BSA | Bovine Serum Albumin |
| PVDF | Polyvinylidene Difluoride |
| HRP | Horseradish Peroxidase |
| FPKM | Fragments Per Kilobase of transcript per Million mapped reads |
| TPM | Transcripts Per Million |
| TMB | Tumor Mutation Burden |
| ANOVA | Analysis of Variance |
